# Cryptic or pseudocryptic: can morphological methods inform copepod taxonomy? An analysis of publications and a case study of the *Eurytemora affinis* species complex

**DOI:** 10.1002/ece3.1521

**Published:** 2015-05-25

**Authors:** Dmitry Lajus, Natalia Sukhikh, Victor Alekseev

**Affiliations:** 1 Saint-Petersburg State UniversitySaint-Petersburg, Russia; 2 Zoological Institute of Russian Academy of SciencesSaint-Petersburg, Russia

**Keywords:** Cryptic species, *Eurytemora affinis*, *Eurytemora carolleeae. Eurytemora caspica*, fluctuating asymmetry, morphological variation, principal component analysis, pseudocryptic species

## Abstract

Interest in cryptic species has increased significantly with current progress in genetic methods. The large number of cryptic species suggests that the resolution of traditional morphological techniques may be insufficient for taxonomical research. However, some species now considered to be cryptic may, in fact, be designated pseudocryptic after close morphological examination. Thus the “cryptic or pseudocryptic” dilemma speaks to the resolution of morphological analysis and its utility for identifying species. We address this dilemma first by systematically reviewing data published from 1980 to 2013 on cryptic species of Copepoda and then by performing an in-depth morphological study of the former *Eurytemora affinis* complex of cryptic species. Analyzing the published data showed that, in 5 of 24 revisions eligible for systematic review, cryptic species assignment was based solely on the genetic variation of forms without detailed morphological analysis to confirm the assignment. Therefore, some newly described cryptic species might be designated pseudocryptic under more detailed morphological analysis as happened with *Eurytemora affinis* complex. Recent genetic analyses of the complex found high levels of heterogeneity without morphological differences; it is argued to be cryptic. However, next detailed morphological analyses allowed to describe a number of valid species. Our study, using deep statistical analyses usually not applied for new species describing, of this species complex confirmed considerable differences between former cryptic species. In particular, fluctuating asymmetry (FA), the random variation of left and right structures, was significantly different between forms and provided independent information about their status. Our work showed that multivariate statistical approaches, such as principal component analysis, can be powerful techniques for the morphological discrimination of cryptic taxons. Despite increasing cryptic species designations, morphological techniques have great potential in determining copepod taxonomy.

## Introduction

Cryptic species are usually understood to be species that are difficult to distinguish using traditional systematics methods (Knowlton 1993), or species that “are classified as a single nominal species because they are at least superficially morphologically indistinguishable” (Bickford et al. 2007). Traditionally, taxonomists have utilized morphological analysis for the description of species, but new genetic methods have significantly increased interest in cryptic species in recent decades (Jörger and Schrödl 2013). Understanding cryptic biodiversity is important for resolving practical conservation questions, in studies of pathogenic organisms, and for addressing theoretical problems of speciation (Bickford et al. 2007). It is also relevant for ecology, particularly for understanding fundamental relation between species and their ecological niches (Marrone et al. 2013).

Different researchers have different opinions about the nature of cryptic species. Some authors consider cryptic species to represent the initial stage of speciation, after newly originated forms have obtained reproductive isolation, but before they have developed detectable morphological differences, that is, cryptic species are evolutionary young forms that are more similar genetically than ordinary species in a group. Other authors consider genetic distances between cryptic species to be similar to distances between ordinary species; they do not represent the initial stage of speciation. Empirical examples may support both opinions. For instance, studies have found that numbers of coccolithophores, considered to be cryptic, are genetically very close to each other (Saez and Lozano 2005; Saez et al. 2008). At the same time, other studies of crustaceans and fish show less genetic similarity among cryptic species (Colborn et al. 2001; Lefebure et al. 2006). In our study, we will not focus on the biological nature of cryptic species, but, rather, consider methodological questions.

There is quite a high probability that many species considered to be cryptic are, in fact, pseudocryptic, that is, they are included in this category because of “the inadequacy of the morphological analysis” (Knowlton 1993). This inadequacy is not because of fundamental limitations of morphological methods, but due to insufficient thoroughness in their application during species description. Careful morphological analysis of species originally considered cryptic, based on morphological similarity in conjunction with genetic, ecological, or behavioral differences, can often establish morphological traits sufficient for distinct identification (Gomez et al. 2004; Dayrat 2005; Saez and Lozano 2005; Will et al. 2005; Cardoso et al. 2009). To find such traits, one may need to study different life stages. This is clearly demonstrated by the butterfly *Astraptes fulgerator,* in which lineages are indistinguishable in the adult stage, but clearly detectable in caterpillars (Hebert et al. 2004). Such cases are more properly termed pseudocryptic species.

Why is it important to differentiate between true cryptic and pseudocryptic species? The existence of true cryptic species shows that morphological analysis has fundamental limitations for discriminating among species. As it is insufficient for describing biodiversity at the species level, nonmorphological techniques such as genetic analysis and investigations of behavioral, physiological, and other traits must be employed. Mayr (1963) assigned great importance to cryptic species (or sibling species in his original terminology) in his attack on the morphological concept of species. However, the existence of pseudocryptic species means that morphological methods may be capable of resolving fine-scale differences among species if their potential is fully utilized. Therefore, the “cryptic or pseudocryptic” dilemma speaks to the resolution of morphological analysis in taxonomical studies, in other words, to the utility of morphological methods for identifying species. According to Knowlton (1993), one might expect that cryptic species are more common in marine environments because it is more difficult to study marine organisms than terrestrial ones. In addition, marine organisms rely on chemical signals for gamete recognition and mate choice, and depend less on vision during reproduction than terrestrial organisms. The copepods selected for this study are typical aquatic organisms possessing these features, and cryptic species are common among them.

We pay special attention to the copepod *Eurytemora affinis* species complex where cryptic species have been intensively studied. *E. affinis* is distributed in brackish waters of the North Pacific and Atlantic Oceans and in some freshwater lake basins. Until recently, most authors considered it a single species (Rylov 1922; Croskery 1978; Dussart and Defaye 2002). However, subsequent genetic studies and the crossbreeding of animals from different regions showed significant genetic differences (Knowlton 1993; Lee 1999, 2000; Lee and Frost 2002; S. Souissi, pers. comm.). Four main clades – European, Asian, North American, and North Pacific – were observed within the species, with maximum pairwise divergences of 10% in 16S rRNA and 19% in COI genes (Lee 2000). Genetic heterogeneity of 01.7–12.4% in COI and 4–6% in 16S rRNA within each clade was also found (Winkler et al. 2008; Winkler et al. 2011). The genetic differences in mitochondrial genes among *E. affinis* clades correspond to species-level differences in *Eurytemora* and in Copepoda in general (Bucklin et al. 1998; Lee 2000; Lefébure et al. 2006; Mcmanus and Katz 2009).

Lee and Frost (2002) carried out a morphological analysis of samples collected from 43 locations throughout their range to describe the main patterns of morphological variation and compare them with genetic variation. The authors concluded that samples of *E. affinis* were significantly more heterogeneous genetically than morphologically. They explained this in terms of morphological stasis within the group and concluded that *E. affinis* represented a complex of cryptic species. However, recently, two separate species *E*. *carolleeae* Alekseev et Souissi and *E. caspica* Sukhikh et Alekseev have been described within the *E. affinis* complex using genetic and morphological techniques (Alekseev and Souissi 2011; Sukhikh and Alekseev 2013). Therefore, this complex represents a convenient model for studying relationships between cryptic and pseudocryptic species.

Along with traditional analysis of mean values of morphological characters, we also studied fluctuating asymmetry (FA) – random deviations from perfect symmetry, a measure of developmental instability (Zakharov 1989), which represents a stochastic component of phenotypic variance (Lajus et al. 2003). FA now is often used for monitoring stress of different origins (Graham et al. 2010; Beasley et al. 2013). This study has two objectives. Because no protocol currently exists to assign cryptic status to a species, we will first analyze the available literature on cryptic species in Copepoda to discover what justifications appear in practical scientific work. Secondly, we will perform a detailed morphological analysis of forms suggested earlier as cryptic species within the *E. affinis* complex, based on trait mean values and principal component analysis, and traditional indices to search for heterogeneity within the groups to determine or confirm their actual status and show robustness and potential of used methods. Also, we compared the samples by fluctuating asymmetry (FA) as an additional morphological marker.

## Materials and Methods

### Terminology

The terms used in relation to morphologically similar forms are diverse and numerous. Whenever possible in this study, we use the term “cryptic species” in its most generic sense. However, we must also mention other terminology. “Twin species” or “sister species” are morphologically similar species with minimal genetic distance between them and sharing a common ancestor unique to them (Borkin et al. 2004; Bickford et al. 2007). “Sibling species” are characterized by greater genetic distances than twin species, distances that are similar to those of “usual” species. “Semispecies” are slightly divergent geographical replacement species that may hybridize infrequently where they overlap (Mallet 2001). Clearly, when quantitative criteria of divergence are absent, these terms are used very subjectively. The term “form” in this study refers to taxa of different or unknown rank. “Clade” is used to emphasize monophyletic origin of a taxon.

### Systematic review of Copepoda cryptic species

A taxonomical revision may produce different results: (1) confirmed status of the “old” form due to insufficient differences between intraspecific forms (if any), (2) subdivision of the “old” species into “usual” species if differences between forms are great, and (3) designation as cryptic species. Although the total number of revisions was notably greater, here, we considered only revisions to the third group.

We analyzed the available data on cryptic species of Copepoda found in literature published in English from 1980 to 2013, performing our last search on 31 January 2014. For public domain Internet and database searches (OvidSP, ScienceResearch, ScienceDirect, eLibrary.ru, Google Scholar, HighWire Press and home pages of scientific publishers Springer, PLOS One, and Blackwell Publishers), we used the keywords “cryptic species,” “twin species,” “sibling species,” “sister species,” “semispecies,” and “clade.” Only publications in peer-reviewed international journals were included.

Initially, the literature search identified 33230 potentially relevant abstracts, from which 100 were retrieved and 24 were included in this review after full examination. Two researchers performed literature searches and data extraction. The first researcher extracted data from listed sources; the second author double-checked this work. Disagreements between researchers were resolved by consensus.

For each publication, we identified the information used to prove that the forms under consideration differed at the species level. Then, we listed any morphological studies that had also been performed. If no morphological analyses had occurred at that time, we identified previous analyses referred to in the study. We used only the authors' terminology. Note that, given the subjectivity of these terms, forms can have different biological natures even when they have the same names.

### Sampling and preservation of the *Eurytemora affinis*

The material for this study was collected from aquatic surface layers (1–2 m deep) using a 100-*μ*m plankton net deployed from a boat or from shore. Most samples were preserved in 95% ethanol, but samples from the Caspian Sea were preserved in a 4% formalin solution.

### Identification of samples

Genetic identification of samples was accomplished using the mitochondrial CO1 gene. In Baltic Sea locations (Gulf of Finland, Gulf of Riga, and the Vistula Lagoon) where different species*, E. affinis* and *E. carolleeae*, coexist, most individuals were identified genetically as described in our previous study (Sukhikh et al. 2013). This identification was based on published data (Lee 1999, 2000; Lee and Frost 2002) where European, American, and Asian forms were described. We provided comparisons of our data with the published sequences of European and American forms (Alekseev et al. 2009) and deposited sequences in GenBank (HM368364, HM473958–HM474035). In locations where overlap in the ranges of different forms is unknown, we relied on published studies (Lee 2000; Winkler et al. 2008, 2011) where detailed analyses of CO1 gene sequences have been performed.

Based on genetic data, taxonomical keys using morphological traits were used to identify newly described species (Alekseev and Souissi 2011; Sukhikh and Alekseev 2013), and the same keys were then used to identify the rest of the individuals.

### Morphological analyses

Samples used for morphological analysis are described in the Table1. We performed analyses of two datasets using structures of caudal rami, protopodite of the swimming legs 5 and protopodite of the swimming legs 4, that are typically employed in taxonomical studies of *Eurytemora* (Gaviria and Forro 2000; Suárez-Morales et al. 2008; Dodson et al. 2010; Alekseev and Souissi 2011; Sukhikh and Alekseev 2013). Copepod adults were measured under a dissection microscope (Olympus, SZX2, Tokyo, Japan) with an ocular micrometer (5-*μ*m resolution). Only females were used for analyses. Type material of *E. carolleeae* and *E. caspica* was studied in the type collection of the Zoological Institute Russian Academy of Sciences under reference numbers 55052–55054 and 55060–55063.

**Table 1:** Characteristics of *Eurytemora* samples used for morphological analysis

Species	Sampling locations	Geographical coordinates	Sampling Date	Code	Sample size, individuals	Number of analyzed traits
*E. caspica*	Caspian Sea	45°48′N, 49°38′E	Jun 2011	1	19	6
*E. carolleeae*	Chesapeake Bay	39°23′81N, 76°03′32W	Apr 2008	2	13	16
*E. carolleeae*	Gulf of Finland	59^o^24′13 N, 28^o^11′06 E	Jul 2008	3	14	16
*E. carolleeae*	Gulf of Finland	59^o^24′13 N, 28^o^11′06 E	Aug 2009	3	31	6
*E. affinis*	Elbe estuary	53^o^53′24 N, 09^o^08′44 E	Mar 2006	4	17	6
*E. affinis*	Seine estuary	49^o^N, 00^o^W	May 2008; Jul 2008	5	17	16
*E. affinis*	Gulf of Riga	57^o^04′44 N, 23^o^04′44 E	Aug 2008	6	28	6
*E. affinis*	Gulf of Finland	59^o^24′13 N, 28^o^11′06 E	Jul 2008	7	14	16
*E. affinis*	Gulf of Finland	59^o^24′13 N, 28^o^11′06 E	Aug 2009	7	31	6
*E. affinis*	Vistula Lagoon	54^o^65′02 N, 20^o^23′37 E	Oct 2007	8	30	6
*E. affinis*	Loire estuary	47^o^17′23 N, 02^o^01′52 W	Jul 2009	9	4	6
*E. affinis*	Gironde estuary	45^o^31′00 N, 01^o^57′00 W	Mar 2005; Apr 2006	9	14	6

**Figure 1:**
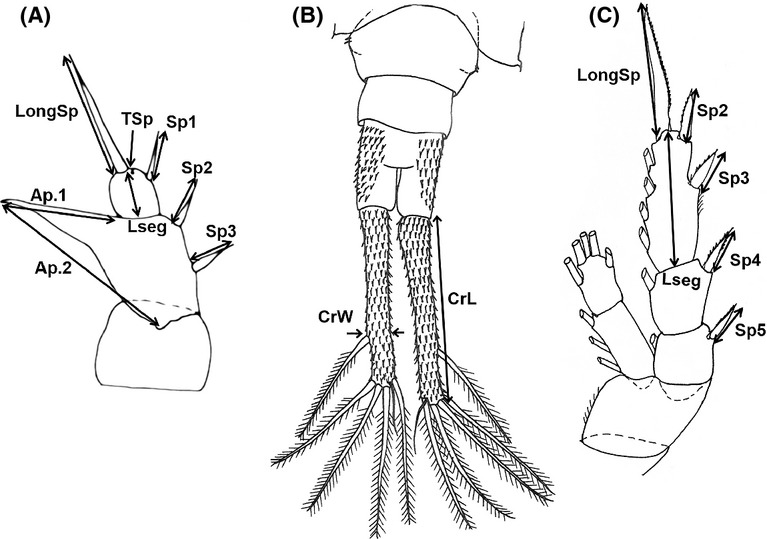
Traits used for the comparison of *Eurytemora* at the rudimentary fifth pair of legs P5 (A), caudal rami (B), and fourth swimming pair of legs P4 (C). Boundaries of traits measurements are indicated with arrows. The six following traits – CrL, FrW (on caudal rami LongSp, Sp1,Sp2, Sp3 (on P5) – were used for analysis of the first dataset (231 specimens); all 16 traits were used only for the second dataset (58 specimens) (Table1). Pictures reworked from Sukhikh and Alekseev 2013.

The first dataset was analyzed to obtain an overall picture of the morphological heterogeneity of the *E. affinis* species complex. We used 6 traits – CrL, CrW (on caudal rami), LongSp, Sp1, Sp2, and Sp3 (on P5) (Fig.1) – and included 231 specimens from nine populations and three species – *E. affinis, E. carolleeae,* and *E. caspica*. Samples collected at the same location in different years were pooled. Also, we pooled samples from the Loire River and Gironde estuaries based on their morphological similarity and geographical proximity.

The second dataset was used for in-depth morphological comparison of two species *E. affinis* and *E. carolleeae* described by Alekseev and Souissi (2011), which coexist in the Gulf of Finland (Table1). Analysis involved 58 specimens, and each species was represented by two populations. The number of traits was expanded to 16 (Fig.1). This comparison primarily focused on different species (former cryptic species), while the first dataset focused on the groups from different localities. Also, the larger set of traits in the second dataset allowed us to study variation in traditional indices. Our morphological analyses include specimens used in previous studies (Alekseev et al. 2009; Alekseev and Souissi 2011; Sukhikh and Alekseev 2013; Sukhikh et al. 2013) and specimens not analyzed before. In contrast with previous studies, however, we expanded the number of populations and the morphological traits under consideration.

All the traits were measured from both the left and right sides of body. Multiple traits (average between left and right) were processed using principal component analysis. Pairwise comparisons were performed using the Student *t*-test. Heterogeneity among multiple samples was analyzed using one-way ANOVA. In case of multiple comparisons, we used Bonferroni correction (Rice 1989; Armstrong 2014).

To understand how small sample sizes of 3–5 specimens, which were used in some previous studies (Lee and Frost 2002), may affect the results of morphological discrimination of samples, we performed a simulation using our first dataset. We calculated a number of significant (*P* < 0.05) pairwise comparisons of several PCs for sample size ranging from 2 to 13. For each sample size, we used different number of trials avoiding, as much as possible, use of the same specimens. For sample sizes *N* = 2 and 3, we used five trials, for *N* = 4 – 4 trials, for *N* = 5 – 3 trails, for *N* = 6–10 – 2 trails, and for *N* = 11––13 – one trial. After that, we averaged the results of individual trials and divided the obtained averages by number of significant pairwise comparisons in the initial dataset (*N* = 231) to standardize them among PCs (theoretical maximum for dataset of nine samples is 36). Obtained data were averaged among different PCs.

Analysis of fluctuating asymmetry was performed using techniques developed earlier (Lajus and Alekseev 2000). FA was calculated with the following formula:

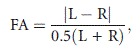
where L and R are the left and right values of the trait, respectively (Lajus and Alekseev 2000). The composite index of fluctuating asymmetry of an individual is the average of the standardized FAs of individual traits. Because distribution of this index is not normal, for statistical comparison of samples by FA, we used Mann–Whitney *U*-test and Kruskal–Wallis ANOVA (Wasserman 2007). Statistica 7.0 and Microsoft Excel 2010 were used for statistical treatment.

## Results

### Cryptic species of Copepoda described in the last three decades

Our literature search showed that during the last three decades, 24 revised copepod species were subdivided into cryptic forms (we use term “form” here because some authors do not assign species status) (Table S1).

Two studies (Conradi et al. 2004; Böttger-Schnack 2005) described sibling species solely based on morphological analysis: The authors interpreted differences between forms to be below the resolution threshold describing ordinary species. Five studies used only genetic techniques (three – only biochemical genetic techniques, one – experimental hybridization, and one – combination of the two methods). In most cases, authors referred to previous studies that showed the absence of morphological differentiation of forms from locations where samples were collected. However, no additional morphological analysis of studied samples was performed. Seventeen studies explored genetic and morphological techniques simultaneously.

### Empirical morphological study of *Eurytemora* species

Overall picture of the morphological heterogeneity of the *E. caspica, E. affinis,* and *E. carolleeae*

We tested normality of distributions of mean values using skewness and kurtosis. Kurtosis and skewness showed significant (*P* < 0.05) departures from normal distribution in 8 and 4 cases, respectively, of totally 54 cases in each dataset. Use of Bonferroni correction resulted in insignificance of all these departures from normality. Based on this, we used parametric statistics in further analysis of mean values.

**Figure 2:**
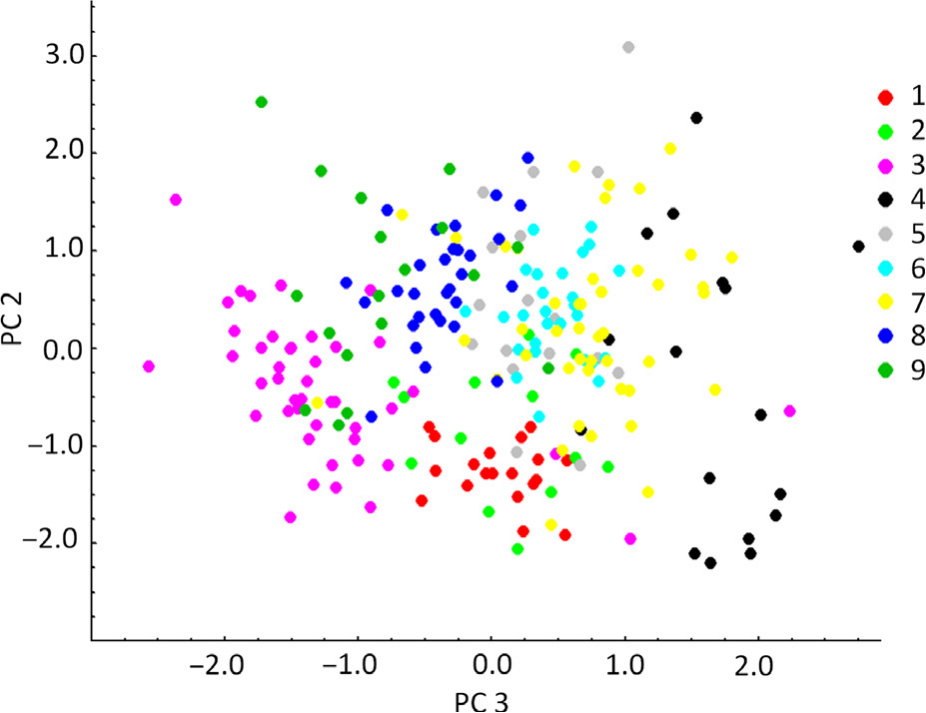
Position of specimens from different samples in coordinates of PC2 and PC3. Numbers of samples are the same as in Table1.

To partition out the effect of size, it is common to apply principal component analysis. Our analyses of 6 traits on 231 specimens from nine samples (Table1) showed that PC1 explained 82.5% of total variance, PC2 – 10.0%, PC3 – 3.2%, PC4 – 2.0, and PC5 and PC6 – 1.1% each. Given that PC1 explained a very high percentage of total variance and that all traits show high loadings on this PC (loadings exceed 0.92 for five traits except length of caudal rami, for which the loading is 0.73), PC1 was interpreted as general size. We interpreted the other PCs as describing different aspects of shape (Bookstein et al. 1985). All PCs except for PC6 showed significant differences between samples (*P* < 0.001) when using one-way ANOVA. These differences remained significant (*P* < 0.01) after Bonferroni correction. This indicated that the samples were differentiated not only by size but also by shape. Discrimination among samples 1–9 by shape is clearly evident when specimens are arrayed against PC2 and PC3 coordinates (Fig.2). Furthermore, three indices – P5Sp2/Sp3, P5Sp1/Sp2, and caudal rami L/W – showed significant differences, also after Bonferroni correction (*P* < 0.01), and the majority of pairwise comparisons of samples are significant: Of 36 pairwise comparisons of 9 samples with each other in PC2 and PC3 by Student's *t*-test, 33 showed significant differences in PC2 and 25 in PC3 (*P* < 0.05). After Bonferroni correction, 17 and 29 comparisons remained significant (*P* < 0.05).

**Figure 3:**
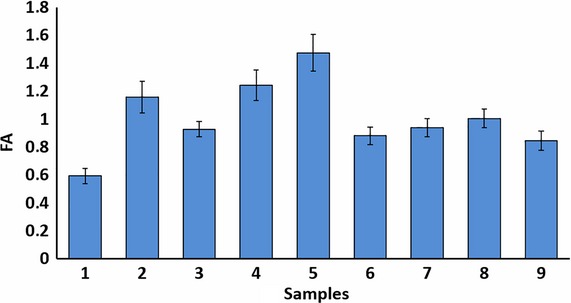
Level of fluctuating asymmetry (FA) calculated as mean of the standardized individual FAs of six traits. Numeration of samples is as in Table1.

Using a *t*-test to compare the left and right values on six traits from four samples (samples 2, 3, 5, and 7) showed no evidence of directional asymmetry. Therefore, FA analysis of the other samples did not differentiate between the left and right sides (i.e., both left and right structures were measured, but were not differentiated). Kruskal–Wallis ANOVA for FA indices of all six traits and for the overall FA index showed significant differences between samples (*P* < 0.01, *P* < 0.05 after Bonferroni correction) (Fig.3). Pairwise comparisons were significant (*P* < 0.05) in 19 of 36 cases. Eleven of them remained significant (*P* < 0.05) after Bonferroni correction.

### In-depth morphological comparison of two species *E. affinis* and *E. carolleeae*

**Figure 4:**
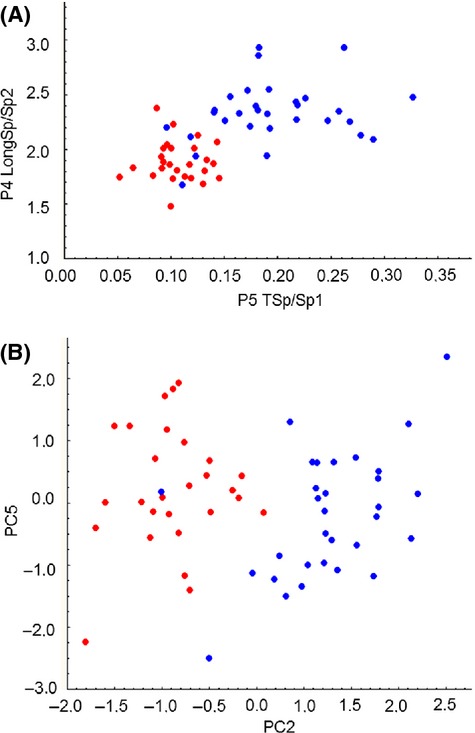
Specimens of *E. carolleeae* from the Gulf of Finland and Chesapeake Bay (red) and *E. affinis* from the Seine River estuary and Gulf of Finland (blue) in coordinates of the most discriminative indices (P5 TSp/Sp1 vs. P4 LongSp/Sp2) (A) and principal components (PC2 and PC5) (B).

Analysis of distribution of mean values of the second dataset did not show significant departures from normality. Kurtosis significantly (*P* < 0.05) departures from normality in two of 20 cases (ten traits that were not included in the first dataset in each sample), but none of them was significant after Bonferroni correction. None of traits showed significant skewness. Analysis of the second dataset showed quite large differences in mean values of the morphological traits. *T*-tests showed that 10 of 16 traits significantly differed at the 95% confidence level, and nine differed at the 99% confidence level (Table2). After Bonferroni correction, 9 and 5 traits showed significant differences for 95 and 99% confidence levels, respectively. Such differences could be interpreted as species-level differences. Traditional indices also demonstrated significant differences between samples, and, in general, differences were more pronounced than among our initial set of traits (Table3). Eight of 10 indices showed significant differences (*P* < 0.01), and Bonferroni correction resulted in shift of *P*-level to *P* < 0.05. While using principal component analysis, significant differences among species were obtained for only PC2, but those were statistically significant at almost any level. Thus, all differences between American and European samples appeared to be aggregated in PC2 (explaining about 10% of total variance). This situation is rare, and in analysis of correlated morphological traits, differences between samples are usually distributed among several PCs (e.g., Lajus and Alekseev 2000). Traits P4LongSp, CrL, and P5TSp had the highest loadings on PC2 (ranging from 0.6 to 0.7). Differences in these traits among samples were also significant, also after Bonferroni correction (*P* < 0.01) (Table2). This suggests that PC2 is not only based on the above three traits but is also correlated with other traits. Discrimination among samples arrayed on coordinates PC2 *vs* PC5 (showing minimal values of *t*-tests) was easier than using other indices that also showed minimal *t*-test values (P5 TSp/Sp1 vs. P4 LongSp/Sp2) (Fig.4A,B).

**Table 2:** Results of comparison of initial traits using Student's *t*-test at different sample sizes and an index of fluctuating asymmetry for 27 specimens of American *E. carolleeae* (sample 2, 3; 2008) and 31 specimens of European *E. affinis* (sample 7, 2009)

Traits	Abbreviation	*P* values of Student's *t*-test (*n* = 31 and 27)	*P* values of Student's *t*-test (*n* = 3)	FA *P* values of Student's *t*-test (*n* = 31 and 27)
caudal rami length	CrL	0.0029	0.4882	0.9015
caudal rami width	CrW	0.0893	0.3690	0.9937
Segment P4 length	Lseg	0.1139	0.7193	0.2259
The longest spine of P4 length	LongSp	0.0028	0.0903	0.0085
Spine 2 of P4 length	P4Sp2	0.3244	0.7451	0.1815
Spine 3 of P4 length	P4Sp3	0.0001	0.3428	0.8528
Spine 4 of P4 length	P4Sp4	0.0018	0.7284	0.7807
Spine 5 of P4 length	P4Sp5	0.0164	0.7600	0.2381
Segment P5 length	P5Lseg	0.0000	0.1537	0.8893
The longest spine of P5 length	P5LongSp	0.0001	0.4028	0.3061
Small spine of P5 length	P5TSp	0.0006	0.3684	0.0924
Spine 1 of P5 length	P5Sp1	0.0000	0.3251	0.7807
Spine 2 of P5 length	P5Sp1	0.0022	0.4669	0.9507
Spine 3 of P5 length	P5Sp3	0.7293	0.7483	0.3855
Appendix 1 of P5 length	P5Ap1	0.5894	0.7476	0.9877
Appendix 2 of P5 length	P5Ap2	0.3027	0.4589	0.1764

Designation of the traits on Figure2.

*T*-test (*n* = 31 and 27) – value of *t*-test at maximal sample size (*n* = 31 and 27).

*T*-test (*n* = 3) – *t*-test value at sample size = 3 (mean for 9 trials using different specimens taken from the initial samples).

*T*-test FA (*n* = 31 and 27) – *t*-test value comparing samples by fluctuating asymmetry (*n* = 31 and 27).

**Table 3:** Values of Student's *t*-test comparing 10 indices at different sample sizes of 27 specimens of American *E. carolleeae* (samples 2, 3; 2008) and 31 specimens of European *E. affinis* (sample 7, 2009)

Index	*P* values of Student's *t*-test (*n* = 31 and 27)	*P* values of Student's *t*-test (*n* = 3)
P5Sp2/P5Sp3	0.0000	0.3972
P5Lseg/P5Sp1	0.0053	0.4578
P5Sp1/P5Sp2	0.0051	0.8512
P5Tsp/P5LongSp	0.0000	0.0861
P5Tsp/P5Sp1	0.0000	0.0398
CrL/CrW	0.0000	0.0662
P4Lseg/P4LongSp	0.1558	0.1474
P4Sp2/P4Sp3	0.1308	0.1792
P4LongSp/P4Sp3	0.0000	0.0248
P4LongSp/P4Sp2	0.0000	0.0121

Abbreviation of the traits in Table2.

*T*-test (*n* = 31 and 27) – value of *t*-test at maximal sample size (*n* = 31 and 27).

*T*-test – *t*-test value (mean for 9 trials using different specimens taken from initial samples).

**Figure 5:**
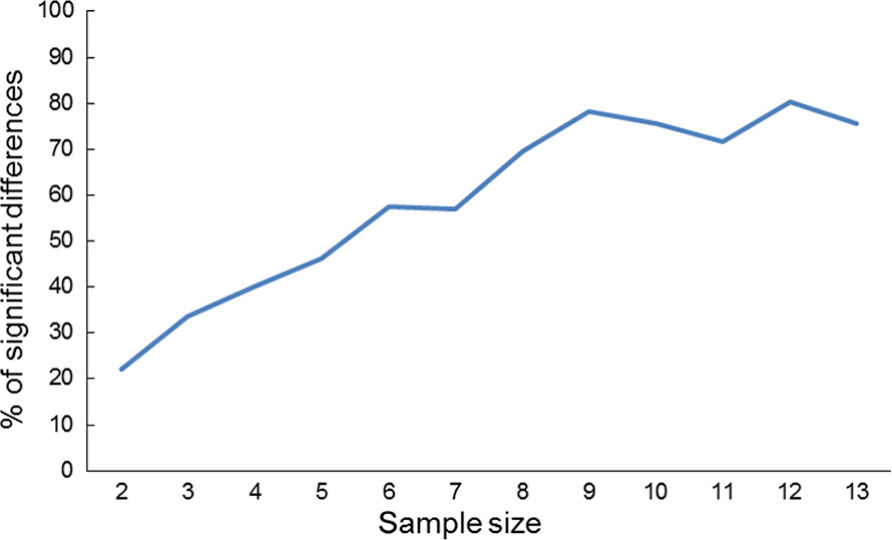
Results of simulation of sample size reduction on significance of differences: percentage of significant (*P* < 0.05) pairwise differences between different *Eurytemora* samples in principal components based on six morphological traits in relation to number of significant differences in the full dataset (9 samples, 231 specimen) averaged for trials and four principal components *vs* sample size (see text for more detailed explanations).

The number of significant pairwise differences, calculated based on simulations using PCs that showed significant effect based on one-way ANOVA (PC2–PC5), clearly shows its dependence on sample size (Fig.5). At sample sizes of 3–5 specimens, the number of significant pairwise comparisons is about 40% of number of significant comparisons while using the full dataset.

In analyzing fluctuating asymmetry, we compared two samples using Mann–Whitney *U*-test and Kolmogorov–Smirnov test and found significant differences in FA (*P* < 0.05) for one of 16 traits (Table2) – long spine 1 (P4). Differences became nonsignificant after Bonferroni correction. At the same time, according to Mann–Whitney *U*-test, in all 16 traits, sum of ranks of individual fluctuating asymmetry of the European sample was higher than the American one, showing statistically significant differences by sign test (*P* < 0.01).

## Discussion

### Cryptic and pseudocryptic species and their relationships with other taxa

**Figure 6:**
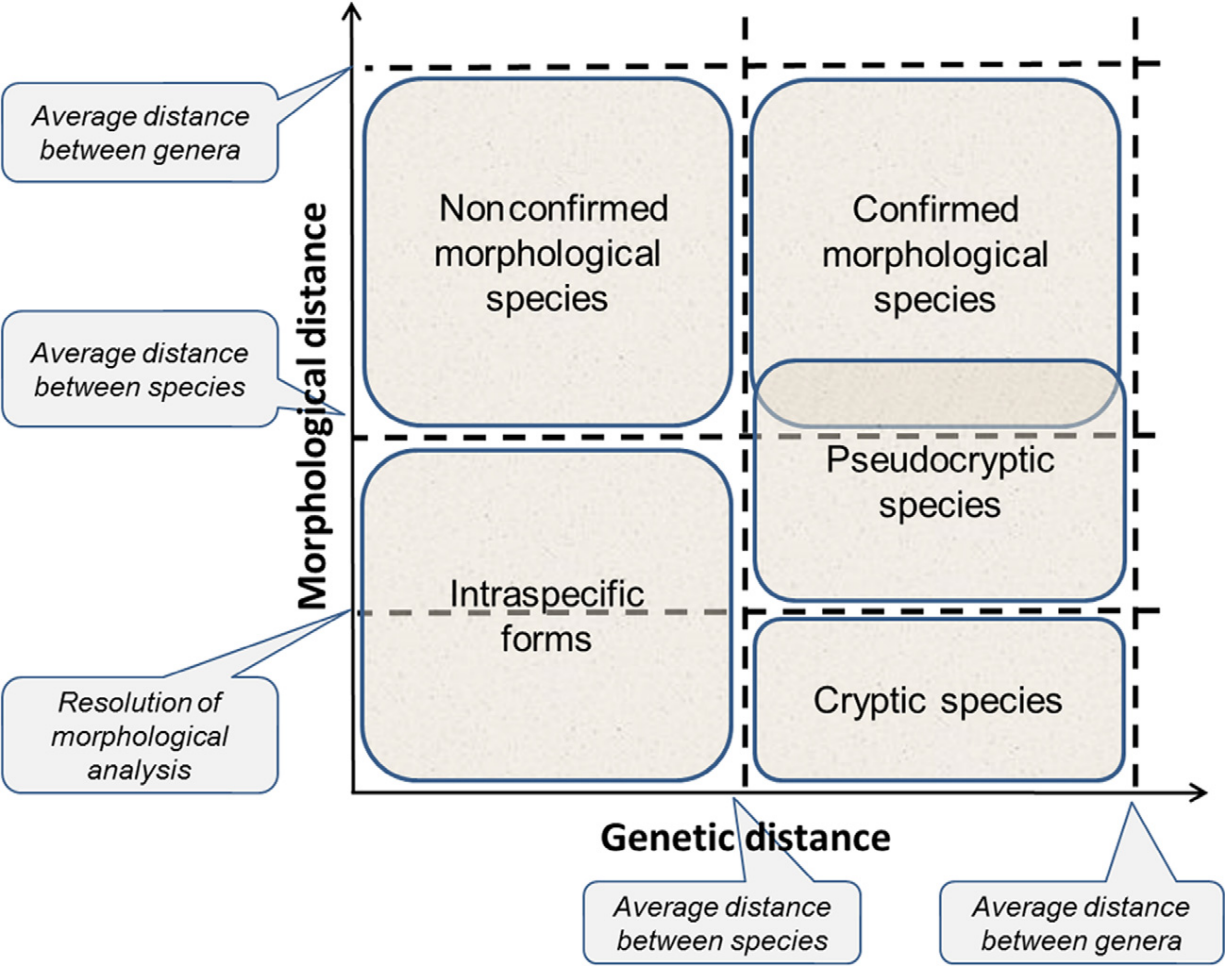
Location of different taxa in coordinates of “genetic similarity” – “morphological similarity.” Explanations are in the text.

To better understand relationships between different taxa, as well as the nature of cryptic and pseudocryptic species, it is useful to represent them graphically as coordinates of genetic and morphological distance. Figure6 is a schematic of this process. The genetic distance axis marks the “average distance between species” and the “average distance between genera.” We do not specify the type of genetic distance, which can differ (Cavalli-Sforza and Edwards 1967; Nei 1972; Reynolds et al. 1983); rather, we use the term broadly, assuming that the average genetic distances between species and genera are known approximately for a particular group. Also, we avoid discussing relationships between genetic distance and reproductive isolation (a key parameter of the biological concept of species), but assume they are correlated. Forms with less genetic separation than is characteristic of species are considered intraspecific groups; those with genetic distances on par with typical genera and species are considered species.

The morphological distance axis has three markers. The first two are “average distance between species” and “average distance between genera.” Much less formalized than genetic distances, these usually result from a consensus among taxonomists working with that particular group of organisms. The third marker, the “resolution of morphological analysis,” is a function of instrumental and statistical error. It may also represent a difference threshold between samples, which could be useful in helping groups of researchers describe intraspecific relationships. For instance, Mann and Evans (2007, p. 248) noted “… some of the differences are so slight that the species are effectively cryptic,” meaning that in some cases, morphological differences can be detectable, but insufficient for assignment as ordinary species.

In this coordinate system, intraspecific forms occupy the lower left-hand corner. To the right are forms first described as species based on morphological analysis, but which genetic analysis did not confirm. The upper right-hand corner is occupied by “usual” species described via morphological analysis and confirmed as such by reproductive isolation or genetic distances. Cryptic and pseudocryptic species are situated in the lower right-hand corner, with pseudocryptic species on top. It is important to stress that pseudocryptic species are above the “resolution of morphological analysis,” as our work has shown.

### How to reduce subjectivity in the assignment of cryptic species status

Our critical analysis of the literature showed that a variety of criteria are used to assign cryptic status to a species. We distinguished three groups of studies. In the first and most common group, 17 of 24 revisions applied an integrative approach combining both genetic and morphological techniques and genetic differences at the species level were compared with minor (or absent) morphological differences. This is the soundest way to assign cryptic status.

The second group, 5 of 24 revisions, based determination solely on genetic analysis, relying on morphological results from previous studies, and even on original descriptions that, for most copepods, occurred in the 19th century (Table S1). This is a weaker basis for discrimination because older taxonomical and statistical methods were more primitive and subtle distinctions between species poorly known. This is evident in the many recent revisions that have identified new species through the use of improved morphological and statistical techniques alone. Reviewing the species concept in diatoms, Mann (1999) noted that all species initially referred as cryptic eventually were found to be morphologically distinguishable using in-depth analysis. It seems that the most correct decision, which could be based on genetic studies without morphological analysis, is to conclude the existence of either cryptic or pseudocryptic species as has been done by Cornils and Held (2014).

The third group included two studies that were based exclusively on detailed morphological analysis and argued that the minor morphological differences observed were not sufficient for status as ordinary species (Boxshall and Self 2011). Broadly speaking, these cases do not contradict existing definitions of cryptic species, which may include criteria that are “difficult to distinguish” (Knowlton 1993) or “at least superficially indistinguishable” (Bickford et al. 2007), but without agreement among experts working with particular forms, such criteria are too subjective and are not distinguishable from resolution of morphological analysis (Fig.6).

Combining morphological and genetic analysis is the best way to study taxon, but even this does not guarantee that a suggested cryptic species is truly cryptic. An example is provided by Rocha-Olivares with co-authors (2001), where cryptic species were supposed as result of huge genetic differences and the first morphological studies showed morphological stasis. However, more deep morphological analysis revealed sufficient differences among studied Harpacticoida and a number of species were described (Gomez et al. 2004). Similar picture was observed in *E. affinis* species complex, which was given a status of cryptic species using integrative approach (Lee and Frost 2002). These examples present that integrative approach by itself is not a guarantee of reliable conclusion due to insufficient use of morphological analysis.

The absence of morphological analysis in the second groups of studies considerably increased the chance for pseudocryptic species status, while the use of only morphological methods made differences between cryptic and ordinary species quite subjective. Thus, in our analysis of published data, the criteria for assigning cryptic status to a species differed by analytical method and cannot be expected to produce consistent results.

Nevertheless, cryptic species are considered a significant component of biodiversity compared with the “elephant in the room” (Adams et al. 2014). Knowledge about cryptic biodiversity is not only an important scientific question but also has great implications for nature management in general and for conservation biology in particular (Witt et al. 2006). Therefore, it is important to standardize as much as possible the procedure of assigning cryptic status to a species. Clearly, combining genetic and morphological analysis in the framework of integrative taxonomy (Dayrat 2005; Will et al. 2005; Cardoso et al. 2009) would reduce the number of pseudocryptic species, whereas abandoning morphological analysis would notably increase chances for eventually changing species status from cryptic to pseudocryptic. On the whole, our examination of cryptic species in Copepoda generally confirmed Knowlton's opinion (Knowlton 1993) about the “inadequacy of morphological analysis” usually performed for the description of cryptic species.

### Pseudocryptic status of *Eurytemora* species

Comparative analysis of *E. carolleeae* and of *E. affinis* showed that the indices have a higher discriminatory power than the initial traits, but lower than PCs generated by principal component analysis. Similar results were obtained in an earlier comparison of three samples of freshwater copepods *Acanthocyclops signifier* Mazepova, 1952, from Baikal Lake (Lajus and Alekseev 2000). As expected, considerable reduction in sample size decreased statistical significance between samples and, for sample sizes close to those used by Lee and Frost (2002), we detected much fewer pairwise statistical differences between samples than in the large samples.

Often traits for morphological analysis in copepods are measured only on one side of the body. This simplifies measurements and analysis but results in loss of information. Firstly, measuring both trait values results in sampling error that is lower than measuring either one or the other (either left or right). The larger the differences between left and right structures and the higher the measurement error, the greater the difference between sampling error based on one or two measurements. In small and difficult to measure copepod structures, measurement error can be quite high (Lajus and Alekseev 2000).

Secondly, analysis of left and right values allows for measuring fluctuating asymmetry which may yield additional information about morphological differences between the forms. Analysis of FA in the first dataset showed pronounced differentiation of samples. This indicates that some factors caused heterogeneity of samples within developmental stability. However, detailed analysis of patterns of asymmetry and their drivers was not the goal of this study. Here, we merely demonstrated that this morphological parameter provides additional independent information pertaining to species description. These results show that fluctuating asymmetry analysis suggests the pseudocryptic status of forms previously considered to be cryptic species by providing additional evidence about their morphological differentiation.

In our study of the *E. affinis* species complex, previously considered to be cryptic (Lee and Frost 2002), we confirmed morphological differences between described species. This supports our conclusion that a detailed morphological analysis should be an essential part of justifying cryptic species. As the morphological analyses that formerly comprised species descriptions were usually performed at a lower resolution than is needed to designate cryptic species, it is necessary to use many different traits as well as samples of reasonable size.

Our analyses showed that it is reasonable to apply other analytical methods in addition to traditional morphological indices. Multivariate statistical techniques may increase the resolution of morphological analysis. Analyzing bilateral traits on the left and right sides reduces sampling error and provides new information on morphological variation – information about developmental stability measured by FA. Combined with the analysis of mean values, FA can be used as an additional morphological marker in population studies of copepods and in the revision of cryptic species status.

## Conclusion

Our critical survey of literature on cryptic species in copepods and detailed morphological analysis of the *E. affinis* species complex suggest that not all species considered to be cryptic are truly cryptic. This affirms that the potential of morphological techniques to contribute insights into taxonomy – even using traditional structures – is still far from its limit. New techniques, in particularly, scanning electron microscopy, can provide an important complementary source of additional characters. How this potential can be met is a broad problem in taxonomy. At a time when the objective need for taxonomists qualified in current methodologies exceeds professional capacity, calls come to invest more resources in this field (Wheeler et al. 2012). Copepods are among species-rich, but small-sized taxa for which the situation is even more difficult than for other groups (Costello et al. 2006). Training taxonomic experts to measure, analyze, and describe such biodiversity requires extensive time and resources. Financial effort is one reason why taxonomists are becoming scarce at some institutions. At the Natural History Museum, London, UK, the number of traditional taxonomists has fallen 12% over the last 15 years due to institutional investments in molecular biological capabilities (Boxshall and Self 2011). Lack of taxonomical expertise, however, cannot be compensated by molecular biological techniques. We agree that the “…notion that anyone with a thermal cycler and DNA sequencer can act as a taxonomist for any group of organism, however appealing the notion might be, is overly optimistic and biologically specious” (Bickford et al. 2007).

## Supporting Information

Table S1 Copepod species separated into cryptic species since 1980 to 2013.

## References

[b1] Adams M, Raadik TA, Burridge CP, Georges A (2014). Global biodiversity assessment and hyper cryptic species complexes: more than one species of elephant in the room?. Syst. Biol.

[b2] Alekseev V, Souissi A (2011). A new species within the *Eurytemora affinis* complex (Copepoda: Calanoida) from the Atlantic Coast of USA, with observations on eight morphologically different European populations. Zootaxa.

[b3] Alekseev VR, Abramson NI, Sukhikh NM (2009). Introduction of sibling species to the ecosystem of the Baltic sea. Dokl. Biol. Sci.

[b4] Armstrong RA (2014). When to use the Bonferroni correction?. Ophthalmic Physiol. Opt.

[b5] Beasley DE, Bonisoli-Alquati A, Mousseau TA (2013). The use of fluctuating asymmetry as a measure of environmentally induced developmental instability: a meta-analysis. Ecol. Ind.

[b6] Bickford D, Lohman DJ, Sodhi NS, Ng PKL, Meier R, Winkler K (2007). Cryptic species as a window on diversity and conservation. Trends Ecol. Evol.

[b8] Bookstein FL, Chernoff B, Elder R, Humphries J, Smith G, Strauss R (1985). Morphometrics in evolutionary biology: the geometry of size and shape change, with examples from fishes.

[b9] Borkin LYA, Litvinchuk SN, Rozanov YUN, Skorinov DV (2004). On cryptic species (exemplified by amphibians). Zool. Zhurnal.

[b10] Böttger-Schnack R (2005). Taxonomy of Oncaeidae (Copepoda, Cyclopoida) from the Red Sea. VII. *Oncaea cristata*, a new species related to the ovalis-complex, and a revision of *O. ovalis* Shmeleva and *O. bathyalis* Shmeleva from the Mediterranean. Cah. Biol. Mar.

[b11] Boxshall GA, Self D (2011). http://www.nerc.ac.uk/research/programmes/taxonomy/documents/uk-review.pdf.

[b14] Bucklin A, Caudill CC, Cooksey KC, Bentley AM (1998). Population genetics and phylogeny of marine planktonic copepods. Molecular approaches to the study of the ocean.

[b17] Cardoso A, Serrano A, Vogler AP (2009). Morphological and molecular variation in tiger beetles of the *Cicindela hyrbida* complex: is an ‘integrative taxonomy’ possible?. Mol. Ecol.

[b19] Cavalli-Sforza LL, Edwards AWF (1967). Phylogenetic analysis: models and estimation procedures. Am. J. Hum. Genet.

[b22] Colborn J, Crabtree RE, Shaklee JB, Pfeiler E, Bowen BW (2001). The evolutionary enigma of bonefishes (Albula spp.): cryptic species and ancient separations in a globally distributed shorefish. Evolution (N Y).

[b23] Conradi M, Megina C, Lopez-Gonzalez P (2004). Sibling species of copepods in association with Mediterranean gorgonians. Sci. Mar.

[b24] Cornils A, Held C (2014). Evidence of cryptic and pseudocryptic speciation in the *Paracalanus parvus* species complex (Crustacea, Copepoda, Calanoida). Front. Zool.

[b26] Costello MJ, Bouchet P, Emblow CS, Legakis A (2006). European marine biodiversity inventory and taxonomic resources: state of the art and gaps in knowledge. Mar. Ecol. Prog. Ser.

[b27] Croskery P (1978). The freshwater co-occurrence of *Eurytemora affinis* (Copepoda: Calanoida) and *Manayunkia speciosa* (Annelida: Polychaeta): possible relicts of a marine incursion. Hydrobiologia.

[b29] Dayrat B (2005). Towards integrative taxonomy. Biol. J. Linn. Soc.

[b32] Dodson S, Skelly D, Lee CE (2010). Out of Alaska: morphological diversity within the genus *Eurytemora* from its ancestral Alaskan range (Crustacea, Copepoda). Hydrobiologia.

[b33] Dussart B, Defaye D (2002). World directory of Crustacea Copepoda of inland waters. I – Calaniformes.

[b37] Gaviria S, Forro L (2000). Morphological characterization of new populations of the copepod *Eurytemora velox* (Lilljeborg, 1853) (Calanoida, Temoridae) found in Austria and Hungary. Hydrobiologia.

[b40] Gomez S, Fleeger JW, Rocha-Olivares A, Foltz D (2004). Four new species of Cletocamptus Schmankewitsch, 1875, closely related to *Cletocamptus deitersi* (Richard) (Copepoda: Harpacticoida). J. Nat. Hist.

[b41] Graham JH, Raz S, Hel-Or H, Nevo E (2010). Fluctuating asymmetry: methods, theory, and applications. Symmetry.

[b43] Hebert PD, Penton EH, Burns JM, Janzen DH, Hallwachs W (2004). Ten species in one: DNA barcoding reveals cryptic species in the neotropical skipper butterfly *Astraptes fulgerator*. Proc. Natl Acad. Sci. USA.

[b500] Jörger KM, Schrödl M (2013). How to describe a cryptic species? Practical challenges of molecular taxonomy. Frontiers in Zoology.

[b46] Knowlton N (1993). Sibling species in the sea. Annu. Rev. Ecol. Evol. Syst.

[b47] Lajus DL, Alekseev VR (2000). Components of morphological variation in baikalian endemial cyclopid *Acanthocyclops signifer* complex from different localities. Hydrobiologia.

[b48] Lajus DL, Graham JH, Polak M, Kozhara AV (2003). Developmental instability and the stochastic component of total phenotypic variance. Developmental instability: causes and consequences.

[b49] Lee CE (1999). Rapid and repeated invasions of fresh water by the copepod *Eurytemora affinis*. Evolution.

[b50] Lee CE (2000). Global phylogeography of a cryptic copepod species complex and reproductive isolation between genetically proximate “populations”. Evolution.

[b51] Lee CE, Frost BW (2002). Morphological stasis in the *Eurytemora affinis* species complex (Copepoda: Temoridae). Hydrobiologia.

[b52] Lefebure TC, Douady J, Gouy M, Trontelj P, Briolay J, Gilbert J (2006). Phylogeography of a subterranean amphipod reveals cryptic diversity and dynamic evolution in extreme environments. Mol. Ecol.

[b53] Lefébure T, Douady CJ, Gouy M, Gibert J (2006). Relationship between morphological taxonomy and molecular divergence within Crustacea: proposal of a molecular threshold to help species delimitation. Mol. Phylogenet. Evol.

[b54] Mallet J, Levin SA (2001). Subspecies, semispecies, superspecies. Encyclopedia of biodiversity.

[b55] Mann DG (1999). The species concept in diatoms. Phycologia.

[b56] Mann DG, Brodie J, Lewis J, Evans KM (2007). Molecular genetics and the neglected art of diatomics. Unravelling the algae the past, present and future of algal systematics.

[b58] Marrone F, Brutto S, Hundsdoerfer AK, Arculeo M (2013). Overlooked cryptic endemism in copepods: systematics and natural history of the calanoid subgenus *Occidodiaptomus* Borutzky 1991 (Copepoda, Calanoida, Diaptomidae). Mol. Phylogenet. Evol.

[b59] Mayr E (1963). Animal species and evolution.

[b60] Mcmanus GB, Katz LA (2009). Molecular and morphological methods for identifying plankton: what makes a successful marriage?. J. Plankton Res.

[b63] Nei M (1972). Genetic distance between populations. Am. Nat.

[b65] Reynolds J, Weir BS, Cockerham CC (1983). Estimation of the coancestry coefficient: basis for a short-term genetic distance. Genetics.

[b66] Rice WR (1989). Analysing tables of statistical tests. Evolution.

[b67] Rocha-Olivares A, Fleeger JW, Foltz DW (2001). Decoupling of molecular and morphological evolution in deep lineages of a meiobenthic harpacticoid copepod. Mol. Biol. Evol.

[b68] Rylov VM (1922). On a new species of Copepoda-Calanoida. Trudy Sankt-Pererburgskogo Obschestva Estestvoispytatelei.

[b69] Saez AG, Lozano E (2005). Body doubles. Nature.

[b70] Saez AG, Zaldivar-Rivero A, Medlin LK (2008). Molecular systematics of the Pleurochrysidaceae, a family of coastal coccolithophores (Haptophyta). J. Plankton Res.

[b72] Suárez-Morales E, Rodríguez-Almaraz G, Gutiérrez-Aguirre MA, Walsh E (2008). The coastal-estuarine Copepod, *Eurytemora affinis* (Poppe) (Calanoida, Temoridae), from arid inland areas of Mexico: an expected occurrence?. Crustaceana.

[b73] Sukhikh NM, Alekseev VR (2013). *Eurytemora caspica* sp.nov. from the Caspian sea – one more new species within the *E. affinis* complex (Copepoda: Calanoida). Proc. Zool. Inst. RAS.

[b74] Sukhikh NM, Souissi A, Souissi S, Alekseev VR (2013). Invasion of *Eurytemora* sibling species (Copepoda: Temoridae) from North America into the Baltic Sea and European Atlantic coast estuaries. J. Nat. Hist.

[b79] Wasserman L (2007). All of nonparametric statistics.

[b80] Wheeler QD, Knapp S, Stevenson DW, Stevenson J, Blum SD, Boom BM (2012). Mapping the biosphere: exploring species to understand the origin, organization and sustainability of biodiversity. Syst. Biodivers.

[b81] Will KW, Mishler BD, Wheeler QD (2005). The perils of DNA barcoding and the need for integrative taxonomy. Syst. Biol.

[b82] Winkler G, Dodson JJ, Lee CE (2008). Heterogeneity within the native range: population genetic an analyses of sympatric invasive and noninvasive clades of the freshwater invading copepod *Eurytemora affinis*. Mol. Ecol.

[b83] Winkler G, Souissi S, Poux C, Castric V (2011). Genetic heterogeneity among *Eurytemora affinis* populations in Western Europe. Mar. Biol.

[b84] Witt JDS, Threloff DL, Herbert PDN (2006). DNA barcoding reveals extraordinary cryptic diversity in an amphipod genus: implications for desert spring conservation. Mol. Ecol.

[b85] Zakharov VM (1989). Future prospects for population phenogenetics. Sov. Sci. Rev. Sect. F. Physiol. Gen. Biol. Rev.

