# Supplementary material for: Cryptic or pseudocryptic: can morphological methods inform copepod taxonomy? An analysis of publications and a case study of the Eurytemora affinis species complex
Source: Ecol Evol. 2015 May 25;5(12):2374–85. doi: 10.1002/ece3.1521 (PMC4475370; doi:10.1002/ece3.1521)
Supplement: Table S1 — Copepod species separated into cryptic species since 1980 to 2013. [file ece30005-2374-sd1.docx]

| Initial species | Constituting forms (species) | Author’s name of forms | Information on species status of forms (genetic data, experiments on hybridization) | Discriminative morphological traits | Reference |
| --- | --- | --- | --- | --- | --- |
| *Eurytemora affinis* (Poppe, 1880) | *E. affinis*; *E. carolleeae* Alekseev et Souissi, 2011; *E. caspica* Sukhikh et Alekseev, 2013; Pacific clade | sibling | COI, 16SrRNA, hybridization | Shape of genital segment, structures of P1, P4, P5 | Lee 2000; Alekseev and Souissi 2011; Sukhikh and Alekseev 2013 |
| *Pseudocalanus spp.* | *Pseudocalanus minutus* (Boeck, 1865); *P. newmani* Frost, 1989; *P. moultoni* Frost, 1989; *P. mimus*  Frost, 1989; *P. acuspes* (Giesbrecht, 1881); *P. major* Sars, 1900; *P. elongatus* (Boeck, 1865) | sibling | 16SrRNA, COl, allozymes | Body size and other stuctures | Bucklin et al.2003; Frost 1989 |
| *Rhincalanus nasutus* Giesbrecht, 1888 | No names | cryptic | 16SrRNA, COI, 18SrRNA, nITS | No indication | Goetze 2003 |
| *Eucalanus hyalinus* (Claus, 1866) | *E. hyalinus*; *E. spinifer* T. Scott, 1894 | sister | 16S rRNA, COI, ITS2 | Various structures | Goetze and Bradford-Grieve 2005 |
| *Acartia tonsa* Dana, 1849 | lineages F (Clade II), S (Clade V); Clades I, III, IV | cryptic | nITS, COI, RFLP COI | No indication | Caudill and Bucklin 2004; Chen and Hare 2008; Costa et al. 2011 |
| *Hemidiaptomus gurneyi* (Roy, 1927) | No names | cryptic | Cyt b | Structure of endopodite P5 | Marrone et al. 2010 |
| *Hemidiaptomus ingens* (Gurney, 1909) | No names | cryptic | Cyt b | Structure of endopodite P5. | Marrone et al. 2010 |
| *Metridia pacifica* Brodsky, 1950 | *M. pacifica;* *M. lucens* Boeck, 1865 | sibling | 16SrRNA | Structure of fifth pair of legs, number of notches on internal margin of the 4^th^ segment | Thorp 1980 |
| *Euchaeta marina*  (Prestandrea, 1833) | *E. marina*; *E. rimana* Bradford,1974 | sibling | 16S rRNA; 28S rRNA | Various structures | Braga et al.1999; Bradford 1974 |
| *Acanthocyclops vernalis* Fisher, 1853 | *A. vernalis;* *A. americanus* Marsh, 1893; *A. robustus* Dodson, 1994 | cryptic | 12S rRNA, COI | Group of spines on upper surface of coxa P4 and two terminal spines on the endopodite P4last segment | Dodson et al. 2003; Blaha et al. 2010; Miracle et al. 2013 |
| *Diacyclops bicuspidatus* (Claus, 1857) | No names | cryptic | hybridisation | No indication | Monchenko 2000 |
| *Diacyclops bisetosus* (Rehberg, 1880) | No names | cryptic | 12S rRNA,  18S rRNA | Various structures | Karanovic and Krajicek 2012a |
| *Macrocyclops albidus* (Jurine 1820) | No names | cryptic | 12S, 16S and cytB | Structures of mandible, аntenne et al.   \| . \|  \| \| --- \| --- \| | Karanovic and Krajicek 2012b |
| *Oncaea ovalis* Shmeleva, 1966 | *O. ovalis*; *O. cristata* Böttger-Schnack, 2005;*O. parabathyalis* Böttger-Schnack, 2005;*O. crypta* Böttger-Schnack, 2005  *O. ovalis* Shmeleva, 1966 | sibling, sister | No indication | Various structures | Dahms et al. 1991 |
| *Tisbe holothuriae* Humes, 1957 | *T. holothuriae*; *T. battagliai* Volkmann-Rocco, 1972; *T.remanei* Volkmann, 1975 | sibling | hybridization | Various structures | Volkmann-Rocco 1972 a,b; Dahms et al. 1991; Chullasorn 2011 |
| *Tisbe inflatiseta* Fava and Volkmann, 1975 | *T. inflatiseta*; *T. bulbisetosa* Volkmann-Rocco 1972 | sibling | hybridization | Various structures | Dahms et al. 1991; Volkmann 1979; Chullasorn 2011 |
| *Tisbe gracilis* (Scott T., 1895) | *T. gracilis*; *T. cucumariae* Humes, 1957 | sibling | hybridization | Various structures | Dahms et al. 1991; Volkmann 1979; Chullasorn 2011 |
| *Tigriopus californicus* Baker, 1912 | No names | semispecies, sibling species | Allozymes, hybridization | No indication | Ganz and Burton 1995; Burton and Lee 1994 |
| *Nannopus palustris* Brady, 1880 | thin-fat/ notched | pseudo-sibling species | Cyt b; 28S rDNA | Shape of a notch on a furca, structures of A2, P3, P4 , | Staton t al. 2005; Garlitska et al. 2012 |
| *Nesippus orientalis* Heller, 1868 | Clades I and II | cryptic/sibling species; pseudo-sibling | COI | No indication | Dippenaar et al. 2010 |
| *Doridicola botulosus* (Stock, Kleeton, 1963) | *D. botulosus*; *D. comai* Conradi, Megina, López-González, 2004 | sibling | No indication | Composition of buccal appendices, shape of 3^rd^ segment of 2^nd^ antenna | Conradi et al. 2004 |
| *Cletocamptus deitersi* (Richard 1897) | *C. deitersi*; *C. deborahdexterae* Gomez, Fleeger, Rocha-Olivares & Foltz, 2004; *C. stimpsoni* Gomez, Fleeger, Rocha-Olivares & Foltz, 2004; *C. sinaloensis* Gomez, Fleeger, Rocha-Olivares & Foltz, 2004; *C. fourchensis* Gomez, Fleeger, Rocha-Olivares & Foltz, 2004 | cryptic | CO1,16S rDNA, nITS, 5.8S rDNA | Number of inner setae on distal segment of the third-swimming leg | Rocha-Olivares et al. 2001 |
| *Pleuromamma piseki Steuer, 1931* | No names | clades | COII | Minimal revision, Characteristics of genital double somite and prosome | Halbert et al. 2012 |
| *Pleuromamma gracilis* (Claus, 1863) | No names | clades | COII | Minimal revision, Characteristics of genital double somite and prosome | Halbert et al. 2012 |

References

Bláha M, Hulák M, Slouková J, Těšitel J (2010) Molecular and morphological patterns across Acanthocyclops vernalis-robustus species complex (Copepoda, Cyclopoida). Zool Scr [39**(**3**)**](http://onlinelibrary.wiley.com/doi/10.1111/zsc.2010.39.issue-3/issuetoc)**:** 259–268.

Bradford JM (1974) *Euchaeta marina* (Prestandrea) (Copepoda, Calanoida) and two closely related new species from the Pacific. Pacif Sci 28: 159-169.

[Braga](http://link.springer.com/search?facet-author=%22E.+Braga%22) E, [Zardoya](http://link.springer.com/search?facet-author=%22R.+Zardoya%22) R, [Meyer](http://link.springer.com/search?facet-author=%22A.+Meyer%22) A, [Yen](http://link.springer.com/search?facet-author=%22J.+Yen%22) J (1999) Mitochondrial and nuclear rRNA based copepod phylogeny with emphasis on the Euchaetidae (Calanoida). [Marine Biology](http://link.springer.com/journal/227) 133(1): 79-

Bucklin A, Frost BW, Bradford-Grieve J, Allen L, Copley NJ (2003) Molecular systematic and phylogenetic assessment of 34 calanoid copepod species of the Calanidae and Clausocalanidae. Marine Biology 142: 333–343.

Burton RS, Lee B-N (1994) Nuclear and mitochondrial gene genealogies and allozyme polymorphism across a major phylo253 genetic break in the copepod *Tigriopus californicus*. Proc natn Acad Sci 91: 5197–5201.

Caudill CC, Bucklin A (2004) Molecular phylogeography and evolutionary history of the estuarine copepod, *Acartia tonsa*, on the Northwest Atlantic coast. Hydrobiologia 511: 91–102.

Chen G, Hare M (2008) Cryptic ecological diversification of a planktonic estuarine copepod, Acartia tonsa. Mol Ecol 17: 1451-1468.

Chullasorn S, Dahms H-U, Lee K-W, Ki J-S, Schizas N, Kangtia P, Park HG, Lee J-S (2011) Description of *Tisbe alaskensis* sp. nov. (Crustacea: Copepoda). Combining Structural and Molecular Traits. Zool Stud 50(1): 103-117 (2011)

Costa KG da, Vallinoto M, Costa RM da (2011) Molecular identification of a new cryptic species of *Acartia tonsa* (Copepoda, Acartiidae) from the Northern coast of Brazil based on mitochondrial COI gene sequences. Journal of Coastal Research (Proceedings of the 11th International Coastal Symposium), Szczecin, Poland, SI 64 p 369-363.

Dahms H-U, HK Schminke, M Pottek (1991). A redescription of *Tisbe furcata* (Baird, 1837) (Copepoda, Harpacticoida) and its phylogenetic relationships within the taxon *Tisbe*. Journal of Zoological Systematics and Evolutionary Research 29:433-449.

Dippenaar SM, Mathibela RB, Bloomer P (2010) Cytochrome oxidase I sequences reveal possible cryptic diversity in the cosmopolitan symbiotic copepod *Nesippus orientalis* Heller, 1868 (Pandaridae: Siphonostomatoida) on elasmobranch hosts from the KwaZulu-Natal coast of South Africa. Exp Parasitol 125(1):42-50.

Dodson SI, Grishanin AK, Gross K, Wyngaard GA (2003) Morphological analysis of some cryptic species in the *Acanthocyclops vernalis* species complex from North America. Hydrobiologia: 500: 131-143.

Frost BW (1989) A taxonomy of the marine calanoid copepod genus Pseudocalanus. Can J Zool 67: 525–551.

Ganz HH, Burton RS (1995) Genetic differentiation and reproductive incompatibility among Baja California populations of the copepod *Tigriopus californicus*. Mar Biol 123(4): 821-827

Garlitska L, Neretina T, Schepetov D, Mugue N, de Troch M, Baguley JG, Azovsky A (2012) Cryptic diversity of the cosmopolitan harpacticoid copepod *Nannopus palustris* genetic and morphological evidence. Mol Ecol 21: 5336-5347

Goetze E 2003. Cryptic speciation on the high seas; global phylogenetics of the copepod family Eucalanidae. Proc Biol Sci 270(1531): 2321-31.

Goetze E, Bradford-Grieve J (2005) Genetic and morphological description of *Eucalanus spinifer* T. Scott 1894 (Calanoida: Eucalanidae), a circumglobal sister species of *E. hyalinus* s. s. (Claus 1866).Prog Oceanogr 65 (1): 55-87.

Karanovic T, Krajicek M (2012a) First molecular data on the Western Australian *Diacyclops*(Copepoda, Cyclopoida) confirm morpho-species but question size differentiation and monophyly of the *alticola*-group. Crustaceana 85 (12): 1549-1569.

Karanovic T, Krajicek M (2012b) When anthropogenic translocation meets cryptic speciation globalized bouillon originates; molecular variability of the cosmopolitan freshwater cyclopoid*Macrocyclops albidus* (Crustacea: Copepoda). International Journal of Limnology 48: 63-80.

Marrone F, Brutto S, Arculeo M (2010) Molecular evidence for the presence of cryptic evolutionary lineages in the freshwater copepod genus *Hemidiaptomu*s G.O. Sars, 1903 (Calanoida, Diaptomidae). Hydrobiologia 644: 115–125.

Miracle MR, Alekseev V, Monchenko V, Sentandreu V, Vicente E (2013) Molecular-genetic-based contribution to the taxonomy of the *Acanthocyclops robustus* group. J Nat Hist 47 (5-12): 863-888.

Monchenko VI *(*2000) Cryptic species in Diacyclops bicuspidatus (Copepoda:Cyclopoida): evidence from crossbreeding studies. Hydrobiologia 417(1): 101-107.

Staton J, Wickliffe L, Garlitska L, Villanueva S, Coull B (2005) Genetic isolation discovered among previously described sympatric morphs of a meiobenthic copepod. J Crustacean Biol 25(4): 551–557.

Thorp A (1980) Comparative morphology of sibling species of *Metridia* (Copepoda: Calanoida). *M. lucens, M. pacifica* and a species indeterminate from Indian arm (British Columbia). PhD thesis. University of British Columbia.

Volkmann B (1979) Tisbe(Copepoda, Harpacticoida) species from Bermuda and zoogeographical considerations. Archivio di Oceanografia e Limnologia 19:1-75.

Volkmann-Rocco B (1972a) *Tisbe battagliain*. sp., a sibling species of *Tisbe holothuriae* Humes ( Copepoda, Harpacticoida). Archivio di Oceanografia e Limnologia 17:259-273.

Volkmann-Rocco B (1972b) Species of *Tisbe* (Copepoda, Harpacticoida) from Beaufort, North Carolina. Archivio di Oceanografia e Limnologia 17:223-258.
